# Interaction between Sex and LDLR rs688 Polymorphism on Hyperlipidemia among Taiwan Biobank Adult Participants

**DOI:** 10.3390/biom10020244

**Published:** 2020-02-05

**Authors:** Yin-Tso Liu, Oswald Ndi Nfor, Lee Wang, Shu-Yi Hsu, Chia-Chi Lung, Disline Manli Tantoh, Min-Chen Wu, Horng-Rong Chang, Yung-Po Liaw

**Affiliations:** 1 School of Medicine, Chung Shan Medical University, Taichung 40201, Taiwan; cvsliu0325@gmail.com; 2 Department of Cardiovascular Surgery, Asia University Hospital, Taichung 40201, Taiwan; 3 Department of Public Health and Institute of Public Health, Chung Shan Medical University, Taichung 40201, Taiwanwl@csmu.edu.tw (L.W.); sui0209@gmail.com (S.-Y.H.); dinoljc@csmu.edu.tw (C.-C.L.); tantohdisline@yahoo.com (D.M.T.); 4 Office of Physical Education, Chung Yuan Christian University, Taoyuan 32023, Taiwan; minchen@cycu.edu.tw; 5 Division of Nephrology, Department of Internal Medicine, Chung Shan Medical University Hospital, Taichung 40201, Taiwan; 6 Department of Medical Imaging, Chung Shan Medical University Hospital, Taichung City 40201, Taiwan

**Keywords:** hyperlipidemia, sex, biobank, polymorphism

## Abstract

Hyperlipidemia is one of the strong risk factors for ischemic heart disease. Using the Taiwan Biobank (TWB) database, we evaluated the risk of hyperlipidemia and its interaction with sex and rs688 polymorphism on the low-density lipoprotein receptor (LDLR) gene. Data collection in the biobank started in 2008 and is ongoing. Data analysis was performed on the participants’ data collected between 2008 and 2015. In general, 27.92% of the 9237 female participants and 32.65% of the 8690 male participants were identified with hyperlipidemia. Compared to the C/C genotype, C/T and T/T genotypes were not significant risk factors for hyperlipidemia (OR = 1.061, CI: 0.976–1.153 for C/T and OR = 1.052, CI: 0.845–1.309 for T/T genotype) in the general model. However, there was a significant interaction between sex and rs6888 on hyperlipidemia risk (p-interaction = 0.0321). With the male sex/CC genotype being the reference group, only the female sex/CT and T/T genotypes were closely associated with hyperlipidemia, with respective ORs of 1.153 (CI: 1.014–1.311) and 1.423 (CI: 1.056–1.917). Our data indicate that rs688 C/T and T/T genotypes may be associated with increased risk of hyperlipidemia in Taiwanese women. These findings may be relevant in lipid-modification therapy.

## 1. Introduction

Hyperlipidemia, defined as elevated total cholesterol (TC ≥ 240 mg/dL), elevated triglyceride (TG ≥ 200 mg/dL), or both, is a well-established risk factor for atherosclerosis, which is closely associated with cardiovascular diseases (CVD) [1,2]. The rates of hyperlipidemia differ with gender, age, socioeconomic status, and ethnicity, and have increased tremendously in the Asia Pacific region over the past decade [3]. This increase in the prevalence of hyperlipidemia has contributed to augmented cardiovascular [4] and cerebrovascular diseases, which are among the major health threats in Taiwan. The prevalence of high TC and high TG in Taiwan were previously estimated at 11.2% and 15.3%, respectively [3]. In another study, age- and sex-specific hyperlipidemia patterns during 1991–2008 reportedly differed greatly among Taiwanese individuals [5].

Several factors ranging from genetic, environmental, and lifestyle factors are associated with hyperlipidemia. Some of its risk factors, such as excessive alcohol intake, smoking, high blood pressures, and others, can be controlled [6]. Secondary or acquired hyperlipidemia occurs frequently and is linked to diabetes, renal disease, and alcoholism [7]. Lifestyle habits and dietary changes are responsible for the increase in the prevalence of hyperlipidemia in Taiwan [5]. Of note, great efforts have been made to control the rate of hyperlipidemia. However, much still needs to be done, especially in high-risk patients [3] since adequate management may serve as an important step to reduce the risk of other conditions linked to hyperlipidemia, such as Alzheimer’s disease [8].

Genetic mutations in several genes play a significant role in the development of hyperlipidemia [9]. Effects of genetic factors on hyperlipidemia may be direct or indirect in that, they may be mediated either by other genetic factors or by environmental and lifestyle choices. The low-density lipoprotein receptor (LDLR) gene is one of those genes that play an important role in maintaining cellular cholesterol homeostasis [10,11]. Several mutations in this gene have been reported to influence exons, splicing sites, and the promoter regions [11], and have also been recognized as a primary cause for familial hypercholesterolemia [12], a condition that has been associated with defective LDL uptake and degradation [4]. Besides, these mutations account for most of the cases with atherosclerosis and coronary heart diseases. One of the LDLR variants, rs688, has shown significant associations with coronary heart disease [13] as well as with higher plasma cholesterol levels (approximately 4%–10%) in different populations [12,14,15,16,17,18]. Furthermore, the rs688 T/T genotype has shown associations with hyperlipidemia [11] and Alzheimer’s disease [19] in a sex-dependent manner. Moreover, it was reportedly associated with higher total and LDL-cholesterol, particularly in pre-menopausal women [12]. Despite these findings, there are no compelling data on the sex-dependent association of rs688 with hyperlipidemia, particularly in Taiwan. Therefore, we evaluated the relationship between rs688 and hyperlipidemia in Taiwanese men and women aged 30–70 years.

## 2. Methods

### 2.1. Study Population and Data Source

The sample consisted of participants who were recruited from 2008 to 2015 into the Taiwan Biobank and whose baseline information was linked to the National Health Insurance Research databases (NHIRD) using encrypted personal identification numbers. Ethical approval was obtained from the Institutional Review Board of Chung Shan Medical University (CS2-16114).

Of the 17,985 biobank participants aged 30–70 years, we excluded those with incomplete information (n = 54) or missing genetic information (n = 4). The final analytic sample included 5416 cases with hyperlipidemia and 12,511 controls individuals.

### 2.2. Definition of Variables

The diagnosis coding in the NHIRD was based on ICD-9 codes. We identified patients with hyperlipidemia (ICD-9-CM: 272), diabetes mellitus (ICD-9-CM: 250), and hypertension (ICD-9-CM: 401–405) who must have had either two outpatient visits or one admission from 1998 to 2015. Alcohol drinkers were defined as persons who reported drinking more than 150 mL of alcohol per week for more than six months. Former drinkers included adults who drank in their lifetime but who had abstained from drinking for over 6 months. Smokers were defined as people who smoked continuously for more than six months. Never smokers included adults who never smoked or did not continuously smoke for 6 months or more while former smokers included those who smoked in their lifetime but who had not smoked in the last 6 months. The body-mass index (BMI) was calculated as weight in kilograms divided by height in meters squared (kg/m^2^). The TC/HDL was obtained by dividing total cholesterol by HDL cholesterol.

### 2.3. Selection of Single Nucleotide Polymorphism

LDLR rs688 was selected based on a literature search. We restricted to this variant because of its significant associations with plasma cholesterol in different populations as cited above. Genotyping in the Taiwan Biobank was performed by Affymetrix using the Axiom™ Genome-Wide Array Plate System (Affymetrix, Santa Clara, CA, USA). Following the Taiwan Biobank (TWB) quality control, we excluded SNPs with low call rates (< 95%) and those that deviated from the Hardy–Weinberg equilibrium (that is, *p* < 1.0 × 10^−3^). We further excluded those with minor allele frequency < 0.05.

### 2.4. Statistical Analysis

Chi-square tests were used to compare the differences between the discrete variables. Logistic regression analysis was used to investigate the effects of sex and LDLR (rs688) gene variants on hyperlipidemia. The odds ratios (ORs) with their 95% confidence intervals were estimated. Statistical analyses were performed using Statistical Analysis System (SAS) software (version 9.4) and PLINK 1.09 beta.

## 3. Results

Table 1 shows the descriptive data by gender. A total of 27.92% of the 9237 female participants and 32.65% of the 8690 male participants were identified with hyperlipidemia. The age groups differed significantly in men and women (*p* < 0.005). Compared to the C/C genotype, C/T and T/T genotypes were not significant risk factors for hyperlipidemia (OR = 1.061, CI: 0.976–1.153 for the C/T and OR = 1.052, CI: 0.845–1.309 for the T/T genotype) in the general model, as shown in Table 2. Compared to women, the odds ratio of hyperlipidemia in men was 0.919 (0.837–1.009). Age was a significant risk factor for hyperlipidemia. The ORs were 1.935 (CI: 1.697–2.207) in the 40–49 age group, 3.975 (CI: 3.495–4.520) in the 50–59 age group, and 6.123 (CI: 5.333–7.030) in the 60–70 age group, respectively; also associated with a higher risk of hyperlipidemia was overweight (OR, 1.281, CI: 1.168–1.404), obesity (OR, 1.557, CI: 1.401–1.730), diabetes (OR, 5.696, CI: 5.099–6.363), hypertension (OR, 3.352, CI: 3.069–3.662), and TC/HDL ≥5 (OR, 1.897, CI: 1.702–2.114). There was a significant interaction between sex and rs6888 on hyperlipidemia risk (p-interaction = 0.0321). Compared to females, the ORs for hyperlipidemia in C/C, C/T, and T/T male carriers was 0.936 (CI: 0.835–1.048), 0.940 (CI: 0.794–1.113), and 0.513 (0.29–0.907), as shown in Table 3. Older age, diabetes, and hypertension remained a significant risk factor no matter the genotype. The association of hyperlipidemia with rs688 genotypes was determined based on sex (Table 4). After stratification by sex, a significant OR was observed only in females with the T/T genotype (OR, 1.357, CI: 1.004–1.835). With the male sex/CC genotype as the reference group (Table 5), only the female sex/CT and T/T genotypes were associated with a significant risk of hyperlipidemia, with respective ORs of 1.153 (CI: 1.014–1.311) and 1.423 (CI: 1.056–1.917).

## 4. Discussion

In this study, we evaluated the relationship between rs688 and hyperlipidemia in Taiwanese men and women aged 30–70 years. Our preliminary results showed that of the overall samples, rs688 C/T and T/T genotypes did not show significant associations with hyperlipidemia when C/C genotype was used as the reference genotype. However, we detected an interaction between the rs688 polymorphism and sex on hyperlipidemia (*p*-interaction = 0.0321). Across the three rs688 genotypes, we found significant associations only among rs688 T/T male carriers; that is, the OR for hyperlipidemia was significant only in T/T (OR, 0.5, CI: 0.29-0.907) but not C/T and CC carriers. It is not certain why the rs688 allele with the higher risk associated with hyperlipidemia was significantly lower in our male population. Nevertheless, this may be attributed to rs688 actions on splicing efficiency, which has been shown to significantly decrease in women compared to men [12]. Besides, the observed association may have been affected by other modulation factors. Nevertheless, more replication studies will be needed to support our findings. Of note, prior investigations in Taiwan have shown gender differences in hyperlipidemia, where the risk was relatively higher in men below 50 years of age, but lower in those over 50 years compared to their female counterparts [3]. However, genetic aspects of hyperlipidemia were not described.

According to findings from previous studies, the LDLR rs688 T/T genotype and T allele are associated with lipid traits [20,21] and heightened risk of coronary artery diseases [11,13]. Our data showed that the risk of hyperlipidemia was significantly lower in men with the T/T genotype as noted above. When men and women were analyzed separately, only T/T women were found to have higher odds of hyperlipidemia. However, when the women and men were analyzed for the three rs688 genotypes, results indicated that T/T and C/T genotypes were significant risk factors in women. In a previous study, cholesterol values for rs688 C/T and T/T carriers were both significantly higher than the C/C carriers, but not different from each other [12]. Associations have also been reported between the T allele of the rs688 polymorphism and elevated total and LDL-cholesterol, particularly in pre-menopausal women [11,12]. It has been established that the rs688 C/T allele would greatly alter LDLR exon 12 splicing efficiency in women, especially those considered to be menopausal [11]. This in part may be the reason why the T allele was the significant risk allele for hyperlipidemia among women in our study.

Previous findings by Guize and colleagues provided evidence that cholesterolemia is closely associated with age and sex [13]. Our study also provided additional information that hyperlipidemia risk increased with age no matter the rs688 genotype. The highest odds ratios occurred in individuals aged 60–70 years. However, also associated with hyperlipidemia was overweight, obesity, and hypertension. These findings share a number of similarities with those of Yin et al. [22], where hyperlipidemia was positively associated with the body-mass index and hypertension in middle-aged and elderly Han and Bai individuals. In contrast, they found no significant relationship between hyperlipidemia and age. However, in their study, stratifications were not made for age. We also demonstrated that hyperlipidemia was closely related to diabetes in all genotype carriers.

We acknowledge that our study may have some limitations. Cholesterolemia has reportedly decreased significantly in women beyond 70. However, our study was restricted to participants aged 30–70, hence we could not analyze data of those over 70 years of age. Second, response bias is a possibility since information was collected using questionnaires.

In conclusion, we demonstrated that there was an interaction between sex and rs6888 on hyperlipidemia risk. Our data indicate that the rs688 C/T and T/T genotypes may be associated with a higher risk of hyperlipidemia in Taiwanese women when compared to men with the C/C genotype. This information may be used to optimize lipid-modification therapy, which may help to prevent atherosclerosis and its clinical manifestations in (but not limited to) these individuals.

## Figures and Tables

**Table 1:** Descriptive data of the participants by gender.

Variable	Female		Male		
	N	%	N	%	*p*-Value
Hyperlipidemia					<0.001
No	6658	72.08	5853	67.35	
Yes	2579	27.92	2837	32.65	
LDL (rs688)					0.695
CC	6078	65.8	5733	65.97	
CT	2849	30.84	2685	30.90	
TT	310	3.36	272	3.13	
Age					0.005
30–39	2385	25.82	2139	24.61	
40–49	2502	27.09	2311	26.59	
50–59	2513	27.21	2327	26.78	
60–70	1837	19.89	1913	22.01	
Education					<0.001
Elementary school	713	7.72	323	3.72	
Junior and Senior high school	4047	43.81	2942	33.86	
University above	4477	48.47	5425	62.43	
Smoking					<0.001
Never	8748	94.71	4769	51.63	
Former	226	2.45	2079	22.51	
Current	263	2.85	1842	19.94	
Alcohol drinking					<0.001
Never	9006	97.50	7049	76.31	
Former	70	0.76	461	4.99	
Current	161	1.74	1180	12.77	
BMI (kg/m^2^)					<0.001
BMI < 18.5	410	4.44	119	1.37	
18.5 ≤ BMI < 24	5380	58.24	3232	37.19	
24 ≤ BMI < 27	2037	22.05	3136	36.09	
BMI ≥27	1410	15.26	2203	25.35	
Diabetes					<0.001
No	8064	87.30	7419	85.37	
Yes	1173	12.70	1271	14.63	
Hypertension					<0.001
No	7478	80.96	6236	71.76	
Yes	1759	19.04	2454	28.24	
TC/HDL (ratio)					<0.001
<5	8587	92.96	6910	79.52	
≥5	650	7.04	1780	20.48	

**Table 2:** Odds ratios for hyperlipidemia in the study participants.

Variable	OR	95%CI	*p*-Value
rs688 (ref: CC)			
CT	1.061	0.976–1.153	0.162
TT	1.052	0.845–1.309	0.651
Sex (ref: Female)			
Male	0.919	0.837–1.009	0.075
Age (ref: 30–39)			
40–49	1.935	1.697–2.207	<0.001
50–59	3.975	3.495–4.520	<0.001
60–70	6.123	5.333–7.030	<0.001
Education (ref: Elementary school)			
Junior and Senior high school	0.933	0.795–1.094	0.391
University above	0.997	0.848–1.172	0.968
Smoking (ref: Never)			
Former	1.021	0.902–1.155	0.746
Current	0.943	0.823–1.081	0.400
Alcohol drinking (ref: Never)			
Former	1.023	0.824–1.271	0.834
Current	1.016	0.871–1.184	0.844
BMI (ref: 18.5≤BMI<24)			
BMI<18.5	0.661	0.493–0.885	0.006
24≤BMI<27	1.281	1.168–1.404	<0.001
BMI≥27	1.557	1.401–1.730	<0.001
Diabetes (ref: No)			
Yes	5.696	5.099–6.363	<0.001
Hypertension (ref: No)			
Yes	3.352	3.069–3.662	<0.001
TC/HDL (ratio) (ref: <5)			
≥5	1.897	1.702–2.114	<0.001

BMI: Body-mass index (measured in Kg/m^2^); TC: Total cholesterol; HDL-C: High-density lipoprotein cholesterol; OR: Odds ratio; CI: Confidence interval.

**Table 3:** Odds ratios for hyperlipidemia based on rs688 genotypes.

Variable	rs688 (CC)		rs688 (CT)		rs688 (TT)	
	N = 11,811		N = 5534		N = 582	
	OR	95%CI	OR	95%CI	OR	95%CI
Sex (ref: Female)						
Male	0.936	0.835–1.048	0.940	0.794–1.113	0.513	0.29–0.907
Age (ref: 30–39)						
40–49	1.925	1.638–2.262	1.912	1.508–2.426	2.346	1.093–5.032
50–59	3.824	3.264–4.481	4.311	3.422–5.431	3.732	1.745–7.981
60–70	5.880	4.960–6.970	6.430	5.015–8.244	8.782	3.911-19.722
Education (ref: Elementary school)						
Junior and Senior high school	0.970	0.797–1.180	0.875	0.654–1.170	1.000	0.434–2.301
University above	1.006	0.824–1.227	1.006	0.748–1.353	0.873	0.366–2.083
Smoking (ref: Never)						
Former	1.080	0.928–1.256	0.879	0.702–1.102	1.195	0.544–2.624
Current	0.975	0.826–1.151	0.856	0.665–1.101	0.966	0.433–2.156
Alcohol drinking (ref: Never)						
Former	1.041	0.794–1.366	1.108	0.758–1.620	0.456	0.124–1.683
Current	1.072	0.890–1.292	0.844	0.633–1.125	2.115	0.848–5.278
BMI (ref: 18.5≤BMI<24)						
BMI<18.5	0.634	0.436–0.921	0.671	0.404–1.113	1.358	0.366–5.040
24≤BMI<27	1.267	1.131–1.419	1.378	1.168–1.626	0.740	0.421–1.302
BMI≥27	1.518	1.335–1.727	1.646	1.358–1.995	1.732	0.924–3.245
Diabetes (ref: No)						
Yes	5.333	4.656–6.110	6.280	5.14–7.672	10.623	5.125–22.019
Hypertension (ref: No)						
Yes	3.402	3.051–3.792	3.310	2.823–3.880	2.854	1.658–4.912
TC/HDL (ratio) (ref: <5)						
≥5	1.957	1.712–2.236	1.757	1.447–2.134	2.519	1.318–4.815
Sex*rs688, *p* = 0.0321,						

BMI: Body-mass index (measured in Kg/m^2^); TC: Total cholesterol; HDL-C: High-density lipoprotein cholesterol; OR: Odds ratio; CI: Confidence interval.

**Table 4:** Odds ratio for hyperlipidemia based on gender.

Variable	Female		Male	
	N = 9237		N = 8690	
	OR	95%CI	OR	95%CI
rs688 (ref: CC)				
CT	1.105	0.980–1.247	1.015	0.904–1.139
TT	1.357	1.004–1.835	0.798	0.579–1.100
Age (ref: 30-39)				
40-49	2.024	1.649–2.484	1.877	1.578–2.234
50-59	5.062	4.158–6.163	3.215	2.702–3.825
60-70	10.287	8.343–12.684	3.811	3.158–4.600
Education (ref: Elementary school)				
Junior and Senior high school	0.875	0.717–1.067	1.220	0.926–1.608
University above	0.900	0.732–1.108	1.355	1.031–1.780
Smoking (ref: Never)				
Former	0.889	0.590–1.342	1.076	0.944–1.226
Current	1.256	0.888–1.778	0.887	0.765–1.028
Alcohol drinking (ref: Never)				
Former	1.331	0.741–2.388	1.019	0.809–1.282
Current	1.025	0.650–1.615	1.021	0.868–1.201
BMI (ref: 18.5 ≤ BMI < 24)				
BMI < 18.5	0.739	0.527–1.037	0.476	0.254–0.893
24 ≤ BMI < 27	1.242	1.084–1.422	1.273	1.120–1.446
BMI≥27	1.562	1.333–1.831	1.483	1.283–1.713
Diabetes (ref: No)				
Yes	5.984	5.089–7.035	5.621	4.819–6.556
Hypertension (ref: No)				
Yes	2.979	2.605–3.408	3.720	3.306–4.186
TC/HDL (ratio) (ref: <5)				
≥5	1.926	1.587–2.338	1.805	1.584–2.057

BMI: Body-mass index (measured in Kg/m^2^); TC: Total cholesterol; HDL-C: High-density lipoprotein cholesterol; OR: Odds ratio; CI: Confidence interval.

**Table 5:** Odds ratio for hyperlipidemia according to gender and rs688 genotypes.

Variable	OR	95%CI
Sex and rs688 genotype (ref: Male and CC)		
Male and CT	1.017	0.905–1.144
Male and TT	0.779	0.562–1.08
Female and CC	1.041	0.935–1.16
Female and CT	1.153	1.014–1.311
Female and TT	1.423	1.056–1.917
Age (ref: 30-39)		
40-49	1.936	1.697–2.208
50-59	3.971	3.492–4.515
60-70	6.128	5.337–7.035
Education (ref: Elementary school)		
Junior and Senior high school	0.935	0.797–1.096
University above	0.998	0.849–1.174
Smoking (ref: Never)		
Former	1.022	0.903–1.156
Current	0.943	0.823-1.080
Alcohol drinking (ref: Never)		
Former	1.027	0.827–1.275
Current	1.016	0.871–1.185
BMI (ref:18.5≤BMI<24)		
BMI<18.5	0.659	0.491–0.883
24≤BMI<27	1.281	1.168–1.404
BMI≥27	1.558	1.402–1.731
Diabetes (ref: No)		
Yes	5.698	5.100–6.366
Hypertension (ref: No)		
Yes	3.352	3.068–3.661
TC/HDL (ratio) (ref: <5)		
≥5	1.898	1.703–2.115

BMI: Body mass index (measured in Kg/m^2^); TC: Total cholesterol; HDL-C: High-density lipoprotein cholesterol; OR: Odds ratio; CI: Confidence interval.
